# Understanding emerging treatment paradigms in rheumatoid arthritis

**DOI:** 10.1186/1478-6354-13-S1-S3

**Published:** 2011-05-25

**Authors:** Ferdinand C Breedveld, Bernard Combe

**Affiliations:** 1 Department of Rheumatology, Leiden University Medical Center, C1-39, PO Box 9600, 2300 RC Leiden, The Netherlands; 2 Immuno-Rhumatologie, Hopital Lapeyronie, CHU Montpellier, Université Montpellier 1, Montpellier F-34000, France

## Abstract

Treatment strategies for rheumatoid arthritis (RA) will continue to evolve as new drugs are developed, as new data become available, and as our potential to achieve greater and more consistent outcomes becomes more routine. Many patients will find both symptom relief and modest control of their disease with disease-modifying antirheumatic drugs (DMARDs), yet this course of therapy is clearly not effective in all patients. In fact, despite strong evidence that intensive treatment in the early stages of RA can slow or stop disease progression and may prevent disability, many patients continue to be managed in a stepwise manner and are treated with an ongoing monotherapy regimen with DMARDs. There is now a large body of evidence demonstrating the success of treating RA patients with anti-TNF therapy, usually in combination with methotrexate. As a result of the increased use of anti-TNF therapy, treatment paradigms have changed – and our practice is beginning to reflect this change. In the present review, we summarize the salient points of several recently proposed and emerging treatment paradigms with an emphasis on how these strategies may impact future practice.

## Introduction

To evaluate the success of any treatment paradigm, it is critical to define the optimal treatment goals in rheumatoid arthritis (RA) and to determine how achievement can be measured.

### Treatment goals

Untreated inflammation leads to tissue damage; and the longer RA is left untreated, the greater the extent of the damage [1]. As most joint damage is largely irreversible, persistent damage will inevitably result in greater disability [1]. The treatment goals in RA therefore include [2,3]: prevention or control of joint damage; prevention of disease progression; prevention of loss of joint function; a decrease of symptoms (for example, pain and stiffness), and achievement of remission or low disease activity; improvement in quality of life (QoL) and maintenance of lifestyle; achievement of drug-free remission; and rapid control of underlying inflammation.

Diagnosis and treatment of RA early in the disease course provides symptom relief and also prevents long-term structural damage and functional decline [4], with a concomitant improvement in QoL and maintenance of everyday activities of daily living. Considering the accepted concept of early treatment in the disease course, a window of opportunity may exist whereby therapeutic intervention could have a disproportionate impact on outcome, resulting in remission induction and maintenance of response after cessation of treatment [5]. The ultimate goal of treatment is to achieve drug-free remission. Previously, despite the fact that drug-free remission is the ideal outcome of therapy, remission in patients with RA was considered rare and unpredictable at the outset of disease [6]. Five-year data from the Behandel-Strategieën (BeSt) study, however, indicate that 19% of patients who received initial combination treatment with methotrexate (MTX) and infliximab achieved drug-free remission [7] – emphasizing that rapid control of underlying inflammation is critical.

### Measures of disease activity

The acute-phase response, a nonspecific reaction to inflammation, is characterized by an increase in the synthesis of certain plasma proteins by the liver, including C-reactive protein (CRP), haptoglobin, and α_1_-antitrypsin [8]. Measuring alterations in acute-phase proteins is an indirect way of determining the presence and severity of inflammation [9]. The erythrocyte sedimentation rate (ESR) and the CRP level are the most commonly used measures of inflammation in RA [8].

Increased CRP levels are associated with decreased functional ability [10] and with increased disease activity and radiological progression in RA [11,12]. CRP is considered a more specific marker of inflammation than the ESR and also serves as a predictor of functional status and joint damage [13]. Additionally, CRP correlates with response to therapy as CRP levels lower or normalize in RA patients following effective treatment [11]. Although CRP is the accepted marker of inflammation, the ESR can provide useful additional information, particularly regarding disease severity; routine analyses of both CRP and the ESR may therefore be beneficial [13].

In terms of remission, definitions that can be used include the American Rheumatism Association preliminary remission criteria and the defined cut-off points for the disease activity score (DAS), the disease activity score in 28 joints (DAS28), the clinical disease activity index, and the simplified disease activity index [14]. Use of the DAS to evaluate disease activity in RA (as in several of the trials described here) has been extensively validated, and current clinical practice is guided by DAS monitoring [15,16]. This DAS tool was developed decades ago when medications and treatment goals were different. Some suggest that DAS28 remission criteria are not stringent enough, and that cut-off points for low/moderate/high disease activity and remission may in the future need to be lower because of more aggressive RA therapy [16].

Furthermore, a Spanish group recently added to the body of evidence supporting use of ultrasound for quantifying inflammation in RA [17]. Ultrasound with power Doppler can be considered an extension of the clinical examination because it provides direct visualization and assessment of synovitis, which may be considered a surrogate for disease activity. These researchers examined 42 joints in each of 97 patients in remission, and compared the ultrasound findings with results from the DAS28 and the simplified disease activity index in the same patients. Interestingly, 92 of 97 (95%) patients supposedly in remission displayed synovial hypertrophy. They found that the simplified disease activity index was superior to the DAS28 in determining absence of inflammatory activity, and therefore in determining remission [17].

The American College of Rheumatology (ACR) and the European League Against Rheumatism (EULAR) are currently preparing a new definition of remission plus updated recommendations, and both organizations encourage researchers to pursue consensus on a uniform definition that may include imaging modalities [14].

Persistent inflammation in RA leads to cartilage and bone destruction [18]. Inflammation and subsequent radiological progression drive disability in RA [19]. Although the degree of disability varies among patients, it is clear that a proportion of patients have disease that progresses particularly rapidly. In this patient subset, rapid control of inflammation is even more important to prevent accumulation of permanent damage. The key to long-term disease control is therefore achieving prompt and substantial control of inflammation.

### Disease progression is patient specific

It has long been recognized that progression of RA is heterogeneous, which means there is high variability in progression and disease activity among patients. Recognition of patients with rapidly progressing disease is critical to identify candidates where intensive therapy may have the most impact in terms of preventing disease progression and maintaining function. Biological markers exist that may be useful in predicting patients at risk for active, progressive disease. Markers such as CRP and the ESR are extensively used to measure the level or degree of inflammation in patients with RA [20].

The Persistent Inflammatory Symmetrical Arthritis (PISA) scoring system has been used in clinical trials to establish patients with poor prognosis who are likely to have rapidly progressing RA [21]. One point each is awarded for rheumatoid factor positivity, for possession of the shared epitope (HLA-DR1/DR4/DR10), for a CRP level >20 mg/l, for female gender, and for a Health Assessment Questionnaire raw score >4; and two points are awarded for a Health Assessment Questionnaire raw score >11. A PISA score ≥3 indicates a poor prognosis [21]. Although this test is used in clinical trials to identify patients with poor prognoses, it is not clear why the PISA system is not practical for routine clinical practice apart from the shared epitope with all the other measures that are routinely used [22].

In routine clinical practice, several factors have been shown to help predict which patients are at risk for radiological progression [23,24]. These biological markers/ clinical indicators include an elevated ESR and CRP level, evidence of erosion, number of swollen joints, high DAS score, and functional ability using the Health Assessment Questionnaire. Autoantibodies, such as rheumatoid factor and anti-cyclic citrullinated peptide antibodies, also are important predictors of outcome in RA. Although these factors are not definitive, if a patient exhibits these clinical signs then the physician can be confident that, without treatment, the patient is more likely to progress rapidly with disability (Table 1).

**Table 1:** Criteria for identifying the rapidly progressing rheumatoid arthritis patient [5,18-20]

Clinical evidence	Subclinical evidence
Early age of onset	Evidence of erosion on radiograph or MRI (van der Heijde–Sharp score ≥2.6)
Failed two DMARDs in 6 months	Elevated CRP level (≥0.6 mg/dl)
≥4 swollen joints	Elevated ESR (28 mm/hour)
Elevated DAS score (≥4.2)	
Health Assessment Questionnaire raw score >4	

CRP, C-reactive protein; DAS, disease activity score; DMARD, disease-modifying antirheumatic drug; ESR, erythrocyte sedimentation rate; MRI, magnetic resonance imaging. Reproduced with permission from Oxford University Press.

Patients with rapidly progressing disease require immediate and intensive control of inflammation to halt disease. Identification of these patients is therefore important as it offers a greater opportunity to change the course of disease.

## Maximizing treatment success in rheumatoid arthritis

### Classic treatment strategies

**Figure 1:**
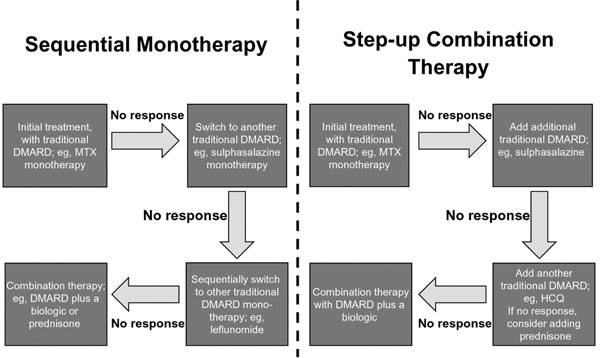
**Traditional treatment paradigms.** DMARD, disease-modifying antirheumatic drug; HCQ, hydroxychloroquine; MTX, methotrexate.

The traditional treatment paradigms in RA are based on one of two approaches: sequential monotherapy or step-up combination therapy (Figure 1) [25].

In sequential monotherapy, treatment is initiated with traditional disease-modifying antirheumatic drug (DMARD) monotherapy, such as MTX. If there is insufficient or no response, patients are switched to mono therapy with another traditional DMARD, such as sulfasalazine [2]. This therapeutic approach may be repeated several times until combination therapy with a DMARD plus a biologic agent, or with a corticosteroid, is introduced as a last resort [2].

In the step-up approach, therapies with the least toxicity are utilized early, and more intensive therapies are added because of lack of response or toxicity. Patients may benefit from consultation with physical or occupational therapists, social workers, and/or patient educators. Nonsteroidal anti-inflammatory drugs or local low-dose systemic steroids may be considered for control of symptoms. The ACR recommends starting treatment with a DMARD within 3 months of diagnosis, whereas the EULAR recommends that DMARD treatment begins as soon as possible [2,26]. DMARDs may be changed or added in patients with inadequate response to treatment (that is, ongoing active disease after 3 months of maximal therapy). Patients who continue to have a suboptimal response may be advanced to receive additional trials of DMARDs, whether used alone or in combination, or may receive treatment with biologic agents [2].

In practice, the frequency of patient visits with a rheumatologist is often determined by disease type. Patients with rapidly progressing disease are prioritized for early review and are seen more often. A pan-European survey of rheumatologists (*n* = 457) established how the rheumatologists identify and treat particular patient types in everyday practice [27]. Forty percent of respondents reported that they see patients with rapidly progressing disease monthly, whereas only 3% see patients with stable disease on a monthly basis. This perceived need for assessment of this group allows rheumatologists to better achieve treatment goals through identification of these patients and use of intensive treatment paradigms such as early use of biologics [27].

Traditional treatments can be suboptimal for patients with RA, because these therapies do not fully address the underlying inflammation driving the disease progression. In particular, response to DMARD monotherapy is frequently suboptimal, and patients with severe RA treated with MTX often exhibit only partial improvement [28]. A significant proportion of patients, however, can attain a state of very low disease activity or remission with DMARDs [29]. Regardless of studies showing DMARD combination therapy to be more effective than monotherapy, single-drug treatment remains the initial treatment approach for most patients [3]. DMARDs have a relatively slow onset of action (1 to 6 months) and may have a less favorable side-effect profile than some other therapies [3,30]. In fact, toxicity is the most common reason for discontinuing treatment with MTX [30]. In addition, conventional DMARDs, even when used intensively, might be less effective in reducing radiographic progression than TNF inhibitors [31]. To meet the RA treatment goals of preventing and controlling joint damage, preventing disease progression and loss of joint function, and improving patients’ QoL, the underlying inflammation of RA must be rapidly suppressed and controlled [2,3].

## Biologic agents: addressing unmet needs

DMARDs alone, including the current gold standard MTX, do not control disease severity, prevent bone and cartilage damage, or maintain QoL in a considerable proportion of RA patients [32,33]. The ideal, most effective treatment should provide rapid and sustained suppression of inflammation, resulting in the maintenance of function and prevention of joint damage [5]. In patients with well-established RA, biologic agents have been shown to effectively improve clinical, functional and radiographic outcomes and to retard radiographic progression [5,34]. Both the ACR and the EULAR, however, currently limit recommendations for addition of a biologic to patients with high disease activity and poor prognosis in whom the DMARD treatment goal was not achieved, and to DMARD-naïve patients with poor prognostic markers [29,34]. As is true for any drug, biologic agents do not achieve optimal response in all patients, and response may diminish over time in some patients.

## Emerging treatment approaches

### Control underlying inflammation to prevent disability and stop disease progression

The first ACR guidelines for the management of RA were developed in 1996 and were subsequently updated in 2002 and 2008 [2,34,35]. Once the initial steps in the management of RA – establishing the diagnosis, performing a baseline evaluation, and estimating the prognosis [2] – are complete, one must move on to consider the optimal treatment strategy for the patient.

The new treatment paradigm recognizes the potential window of opportunity for therapeutic intervention in early disease. Early therapeutic intervention in RA reduces long-term disability and joint damage [6]; the use of the most effective therapy is therefore appropriate in early treatment [5]. Some studies have evaluated the effectiveness of TNF inhibitors (etanercept, adalimumab, infliximab) in early RA [4,5,36,37].

In the double-blind Combination of Methotrexate and Etanercept in Active Early Rheumatoid Arthritis study, patients with early RA (disease duration, 3 to 24 months) were randomized to receive either MTX plus etanercept (50 mg/kg) combination therapy or MTX monotherapy [36]. At 52 weeks, 50% (95% confidence interval (CI), 44 to 56%) of patients in the combination treatment group achieved clinical remission (DAS28 <2.6) with few swollen or tender joints, compared with 28% (95% CI, 23 to 33%) of patients on MTX monotherapy. Furthermore, 80% (95% CI, 75 to 85%) of patients in the combination treatment group and 59% (95% CI, 53 to 65%) of patients in the MTX group achieved radiographic nonprogression (modified total Sharp score change ≤0.5). These results suggest that in addition to achieving an immediate improvement in disability, longer-term disability may be preventable by inhibiting radiographic progression. The study also suggests that remission is an achievable goal in patients with early severe RA within the first year of treatment with etanercept plus MTX [36].

The PREMIER study was a randomized, double-blind clinical trial comparing the efficacy of adalimumab (40 mg/kg) plus MTX combination therapy versus MTX monotherapy or adalimumab monotherapy in patients with early RA (disease duration <3 years) [37]. Combination therapy was superior to both adalimumab and MTX mono therapy in all outcomes in the study. At 1 year, 43% of patients receiving combination therapy achieved clinical remission (DAS28 <2.6), compared with 23% and 21% of patients receiving adalimumab mono-therapy and MTX monotherapy, respectively (*P* < 0.001 for both comparisons). Following the second year of treatment, nearly one-half (49%) of patients receiving combination therapy achieved clinical remission, compared with only 25% of patients receiving adalimumab monotherapy and 25% of patients receiving MTX mono-therapy (*P* <0.001 for both comparisons). Patients receiving combination therapy demonstrated a mean increase in total Sharp score of 1.3 Sharp units, compared with 3.0 units in those receiving adalimumab monotherapy (*P* = 0.002) and 5.7 units in those receiving MTX mono-therapy (*P* <0.001) at 1 year. After 2 years of treatment, patients receiving combination therapy continued to have significantly less radiographic progression (mean change, 1.9 Sharp units) compared with those receiving either adalimumab monotherapy (5.5 units) or MTX monotherapy (10.4 units) (*P* <0.001 for both comparisons). The study demonstrates the superiority of combination therapy with adalimumab and MTX over adalimumab monotherapy or MTX mono therapy with respect to achieving clinical remission and stopping disease progression in patients with early, aggressive RA [37].

In a double-blind study, 20 previously untreated patients with early RA (<12 months of symptoms) of poor prognosis (PISA scoring system) received either MTX plus infliximab (3 mg/kg) combination therapy or MTX monotherapy for 12 months [5]. Treatment was discontinued after 12 months and patients were followed for an additional 12 months. Infliximab plus MTX treatment was found to reduce magnetic resonance imaging (MRI) evidence of synovitis and joint damage compared with MTX alone. MRI synovitis scores dropped from 5.5 at baseline to 3.4 at week 14 in the infliximab group, compared with a reduction from 6.2 to 5.9 in the MTX-alone group (*P* <0.05). The difference was maintained after 12 months of treatment (3.8 vs. 6.6, respectively; *P* <0.05). In the infliximab group, rapid improvements in physical function were sustained throughout the 12-month treatment period. Disease activity remained below remission levels in 70% of patients 12 months after the withdrawal of therapy. Similar benefits were seen in QoL. These results suggest that the rapid control of inflammation demonstrated by infliximab confers long-term functional, QoL, and MRI benefits [5].

**Figure 2:**
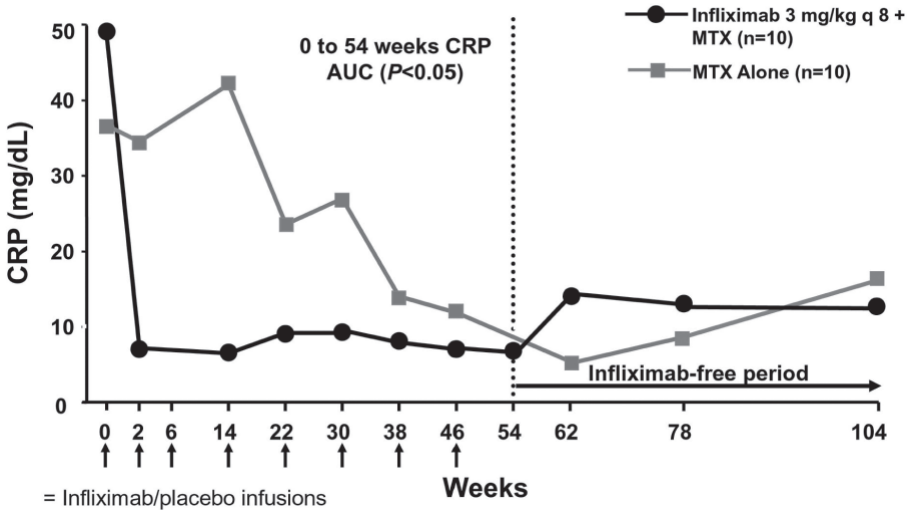
**Infliximab rapidly normalizes C-reactive protein levels in rheumatoid arthritis**[5]. AUC, area under the curve; CRP, C-reactive protein; MTX, methotrexate. Reproduced with permission from [5].

This study by Quinn and colleagues also shows that CRP levels rapidly return to normal levels in patients receiving infliximab combination therapy. Indeed, a single infliximab infusion normalizes mean CRP levels (Figure 2). The reduction in CRP is significantly greater in the infliximab group compared with the MTX-alone group (*P* <0.05) and corresponds with suppression of inflammatory joint disease (MRI synovitis) and resultant prevention of structural damage (MRI erosions) [5].

In the Tight Control of RA study, intensive management with conventional DMARDs, an intra-articular steroid, and frequent clinical assessments was compared with routine outpatient care [31]. The primary outcome measures were a mean decrease in the DAS and in the proportion of patients with a good response. The mean decrease in the DAS was greater in the intensive group than in the routine group (–3.5 vs. –1.9; 95% CI, 1.1 to 1.2; *P* <0.0001). Additionally, patients treated intensively were more likely to have a good response (45/55 (82%) vs. 24/55 (44%); 95% CI, 2.4 to 13.9; *P* < 0.0001) or to be in remission defined by DAS <1.6 (36/55 (65%) vs. 9/55 (16%); 95% CI, 3.9 to 23.9; *P* < 0.001). The Tight Control of RA study showed that a strategy of intensive outpatient management substantially improves disease activity, radiographic disease progression, physical function, and QoL at no additional cost [31].

The concept of using tight control of RA to drive disease-management decisions is also illustrated by the BeSt study [4]. Pisetsky offers some very real conclusions in his ‘provocative and very creative’ [38] landmark randomized clinical trial on treatment strategies for RA [39]. Treatment adjustments were made every 3 months in an effort to obtain low disease activity (DAS in 44 joints (DAS44) ≤ 2.4) and may be described as DAS-driven therapy [7]. Previous data have indicated that treatment with a DMARD such as MTX combined with a TNF inhibitor is more effective than DMARD monotherapy [4]. The anti-TNF therapy (infliximab) used in the BeSt study also has a rapid onset of action (as early as 2 weeks) [2], a favorable safety profile, and sustained effects in many RA patients [4].

**Figure 3:**
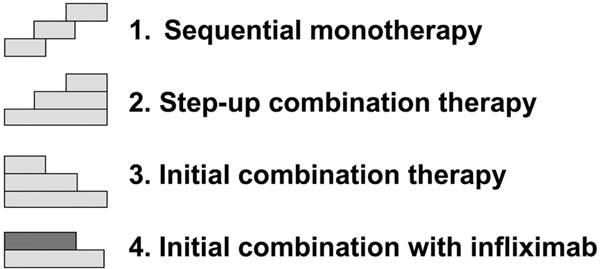
Four treatment strategies of the BeSt analysis [4].

The development of TNF inhibitors presents clinicians with effective treatment options. The increase in therapeutic choices, however, leaves open the question of what is the optimal therapeutic strategy in patients presenting with RA. In an attempt to answer this question, the BeSt study compared the clinical and radiographic outcomes of four different treatment strategies: sequential mono-therapy; step-up combination therapy; initial combination therapy with tapered high-dose prednisone; and initial combination therapy with infliximab (Figure 3). The common goal in all strategies was to rapidly and effectively reduce disease activity by tight monitoring and immediate adjustment of therapy in the case of an insufficient response [4]. The BeSt study is the first in which decisions about changing the dosage or discontinuing infliximab treatment were dictated by DAS calculations before every infusion [40]. It is possible that some conclusions drawn from this study may be extrapolated to all TNF inhibitors.

**Figure 4:**
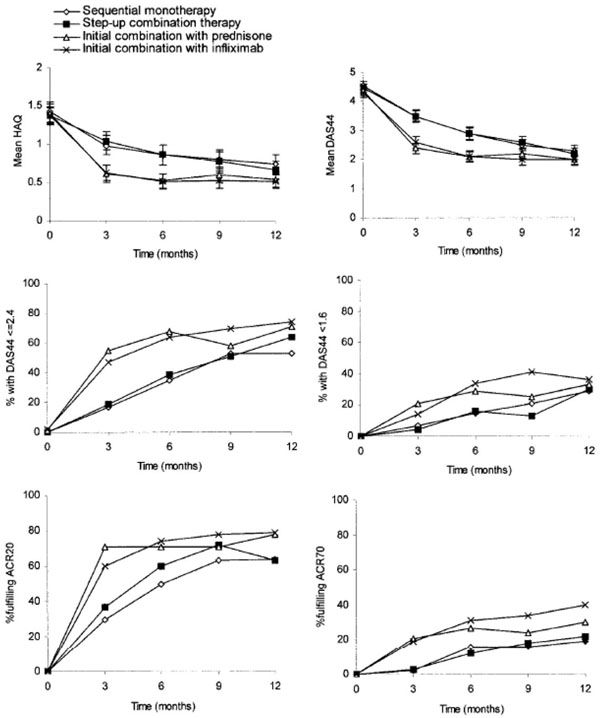
**Clinical outcomes in the BeSt study.** Error bars indicate 95% confidence intervals. DAS44, disease activity score in 44 joints (DAS44 ≤2.4 indicates adequate clinical response; DAS44 <1.6 indicates clinical remission). ACR20/ACR70, 20%/70% improvement according to the American College of Rheumatology response criteria [4]. HAQ, Health Assessment Questionnaire. Reproduced with permission from [4].

The specific objective for each treatment group in the BeSt study was to reach and sustain a DAS44 ≤2.4, indicating low disease activity. After 1 year, this goal was attained by 53% of patients on sequential monotherapy (*P* = 0.004 vs. prednisone; *P* = 0.001 vs. infliximab), by 64% on step-up combination therapy, by 71% on initial combination therapy with tapered high-dose prednisone, and by 74% on initial combination therapy with infliximab (Figure 4) [4].

Patients treated with initial combination therapy, with either prednisone or infliximab, had greater and more rapid functional improvement than patients treated with sequential monotherapy or step-up combination therapy. Additionally, clinical improvement measured by ACR response criteria was achieved earlier and by a greater number of patients treated with initial combination therapy than in the other two groups [4].

After 1 year, patients treated with initial combination therapy had significantly less progression of radiographic joint damage (prednisone, 87%; infliximab, 93%) than those treated with sequential monotherapy (67%) or step-up combination therapy (73%) (*P* < 0.001 sequential vs. prednisone and infliximab; *P* = 0.010 step-up vs. prednisone; *P* < 0.001 step-up vs. infliximab; *P* = not significant for other comparisons) [4].

The patients in each group did not necessarily remain in their initial treatment protocol since treatment was adjusted every 3 months in patients who did not reach and sustain DAS44 ≤2.4. In the sequential monotherapy group, approximately 50% of patients required treatment adjustment and 67% of patients had no progression or radiographic joint damage, suggesting that the initial time of 3 months on MTX monotherapy is too long to prevent erosion. Conversely, 93% of patients receiving infliximab initially have no progression of joint damage [4].

**Figure 5:**
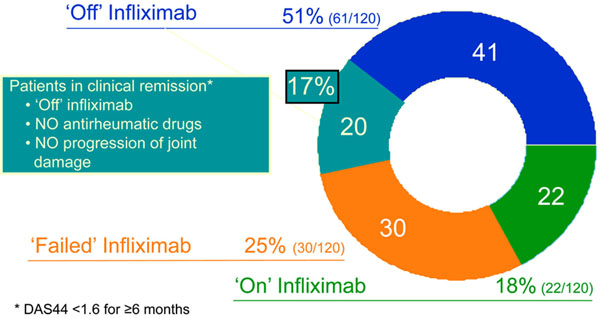
**Some patients enter complete remission with combination therapy**[41]. DAS44, disease activity score in 44 joints.

**Figure 6:**
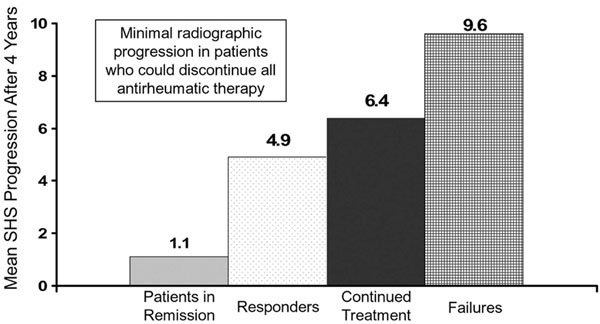
**Less radiographic progression with infliximab combination therapy**[41]. SHS, Sharp–van der Hiejde score.

Four years after starting combination treatment with infliximab and MTX, 51% (61/120) of the patients with very early RA had discontinued infliximab and still had DAS44 ≤2.4. Of these 61 patients that were off infliximab treatment at year 4, 17% (20/120) remained in clinical remission (DAS44 <1.6 for ≥6 months) after stopping all antirheumatic drugs without showing progression of joint damage (Figure 5). Additionally, those 17% of patients receiving initial infliximab combination therapy had discontinued all antirheumatic drugs and achieved clinical remission. Radiographic progression was highest in patients who had failed MTX and infliximab treatment, and was minimal in those 20 patients who discontinued all antirheumatic therapy (Figure 6) [41].

At 5 years, up to 51% of patients achieved remission (DAS <1.6) after sequential monotherapy treatment. Of those remissions, 39% of patients remained on initial sequential monotherapy and 81% remained on the initial allocation to combination treatment with infliximab (*P* <0.001 vs. sequential monotherapy). Overall, 48% of all patients achieved remission and up to 19% achieved drug-free remission. Initial treatment of patients with early, active RA with infliximab and MTX offers the opportunity to discontinue infliximab in the majority of patients once a low DAS is achieved and maintained without flare of the disease. Additionally, a low dosage of MTX may maintain a low level of disease activity in most responders [40].

The BeSt study illustrates that early and intensive suppression of RA activity with a TNF inhibitor may provide earlier clinical improvement and less progression of joint damage. Furthermore, the use of initial combination therapy does not result in increased toxicity. Additionally, combination therapy can be withdrawn successfully and less treatment adjustments are needed than with initial monotherapies. This cooperative study in an organized healthcare setting demonstrated that there appears to be an opportunity for patients initially diagnosed with RA to achieve a lasting benefit in the course of their disease. Furthermore, true clinical remission without any continuing or ongoing maintenance therapy may even be possible [42].

In terms of selection of therapy, the most recent ACR guidelines recommend use of TNF inhibitors in DMARD-näive patients with early RA and high disease activity. Combination treatment of a TNF inhibitor plus MTX is recommended if high disease activity is present for 3 to 6 months or for <3 months with features of a poor prognosis (plus reimbursement-related qualifications) [34]. Current EULAR guidelines recommend use of TNF inhibitors when the initial DMARD-alone strategy has failed and poor prognostic factors (that is, autoantibodies, high disease activity, early erosions) are present [29].

Data from the BeSt study indicate that initial treatment with infliximab plus MTX results in significantly better functional ability over 5 years than other treatment strategies [7]. In addition, infliximab provides rapid disease control with corresponding suppression of inflammatory disease resulting in functional, QoL, and MRI damage benefits [5]. The early use of TNF inhibitors may have a specific effect on the processes that sustain underlying inflammation [5].

As part of any treatment decision process, one must consider both the benefits as well as possible safety risks. Additionally, patient-specific parameters need to be considered when selecting a treatment strategy. One such treatment strategy can include early intensive treatment with biologic therapy. There are safety considerations with the use of biologics such as TNF inhibitors, which include risk of infections, lymphoma, and infusion-site or injection-site reactions. Patients should be monitored regularly for potential safety issues while being treated with a TNF inhibitor.

In the BeSt study, no significant differences were found in the number of adverse events and withdrawals between the groups during the first 12 months [4]. In particular, no cases of tuberculosis or opportunistic infections were reported [4]. In clinical studies with infliximab, adverse reactions are observed in approximately 60% of infliximab-treated patients and 40% of placebo-treated patients. Infusion-related reactions are the most common adverse reactions reported, and dyspnea, urticaria, and headache are the most common causes of discontinuation [43].

Clinical practice is beginning to change as a result of a paradigm shift that incorporates the increased use of TNF inhibitor treatment strategies. Optimal treatment of patients with RA requires comprehensive coordinated care and the expertise of a number of healthcare providers. Proper identification of patients with active, progressive disease is important; early intervention therefore offers a tremendous opportunity to change the course of disease and avoid the serious consequences of disease progression [2,26]. At each follow-up visit, the disease must be assessed as being active or inactive. Tracking disease progression can be difficult, and patients at risk for active, progressive disease may go undetected. Occasionally, joint examination alone may not adequately reflect disease activity and structural damage; periodic measurements of other factors that may be predictive of radiological progression can therefore be assessed in routine clinical practice. Such factors include the ESR or CRP level and functional status as well as radiographic examinations of involved joints. Intensive treatment before the onset of joint damage and disability has the ability to improve patient outcomes and may prevent irreversible joint damage [2,26].

Equally important is identification of patients who will respond optimally to biologic agents and/or to early intensive therapy. Evidence sufficient to support predictors of response, however, is lacking [29,44,45].

## Conclusions

The underlying inflammation that is critical for disease progression in chronic inflammatory diseases such as RA is not fully treated with traditional therapies. As conventional nonbiologic monotherapy is the first treatment offered to the majority of patients, it is clear that most patients are suboptimally or undertreated for RA. Important treatment goals in RA patients include achieving remission, prevention and control of joint damage, avoidance of further disease progression and loss of joint function, and improvement in QoL. In order to meet these challenges, the underlying inflammation of RA must be rapidly suppressed and controlled [2,3].

Accumulating data show that intensive treatment strategies with biologic agents, especially the TNF inhibitors (that is, infliximab), is more effective than sequential monotherapy or step-up combination therapy and should be adopted early in the course of RA [4]. TNF inhibitors show substantial efficacy in combination with MTX, providing rapid and substantial benefit and improvement in patient outcomes.

In addition to the importance of understanding the efficacy and safety profile of a particular drug in RA, recognizing the optimal treatment paradigm in which that drug fits is equally essential. Times are changing, and both our understanding of disease processes and the availability of new therapies also drive us to change our practice. In everyday practice, we now have ways to identify those patients who are at risk for more rapid disease progression and the ability to choose to treat these patients more intensively with biologic therapy. In doing this, the evidence suggests we will best prevent the long-term disability effects of RA for our patients.

## Abbreviations

ACR: American College of Rheumatology; BeSt: Behandel-Strategieën; CI: confidence interval; CRP: C-reactive protein; DAS: disease activity score; DAS28: disease activity score in 28 joints; DAS44: DAS in 44 joints; DMARD: disease-modifying antirheumatic drug; ESR: erythrocyte sedimentation rate; EULAR: European League Against Rheumatism; MRI: magnetic resonance imaging; MTX: methotrexate; PISA: Persistent Inflammatory Symmetrical Arthritis; QoL: quality of life; RA: rheumatoid arthritis; TNF: tumor necrosis factor.

## Competing interests

FCB has received speaker fees from Pfizer, Abbott, Centocor, Wyeth and Schering-Plough. BC has been a speaker and/or consultant for Abbott, BMS, MSD, Roche, Schering-Plough, UCB and Wyeth.
